# Metabolic reprogramming of inner ear cell line HEI-OC1 after dexamethasone application

**DOI:** 10.1007/s11306-021-01799-y

**Published:** 2021-05-24

**Authors:** Michel Kather, Sabine Koitzsch, Bernhard Breit, Stefan Plontke, Bernd Kammerer, Arne Liebau

**Affiliations:** 1 Centre for Integrative Biological Signalling Studies CISA, University of Freiburg, Habsburger Straße 49, 79104 Freiburg, Germany; 2 Hermann Staudinger Graduate School, University of Freiburg, Hebelstr. 27, 79104 Freiburg, Germany; 3 Department of Otorhinolaryngology-Head and Neck Surgery, Martin Luther University Halle-Wittenberg, Ernst-Grube-Straße 40, 06120 Halle (Saale), Germany; 4 Institute of Organic Chemistry, University of Freiburg, Albertstraße 21, 79104 Freiburg, Germany; 5 Centre for Biological Signalling Studies BIOSS, University of Freiburg, Schänzlestraße 18, 79104 Freiburg, Germany; 6 Center for Biosystems Analysis, ZBSA, University of Freiburg, Habsburgerstr. 49, 79104 Freiburg, Germany

**Keywords:** Inner ear, Drug delivery, Cochlear implant, GC–MS, Untargeted profiling

## Abstract

**Introduction:**

One approach to dampen the inflammatory reactions resulting from implantation surgery of cochlear implant hearing aids is to embed dexamethasone into the matrix of the electrode carrier. Possible side effects for sensory cells in the inner ear on the metabolomics have not yet been evaluated.

**Objective:**

We examined changes in the metabolome of the HEI-OC1 cell line after dexamethasone incubation as a cell model of sensory cells of the inner ear.

**Results and Conclusion:**

Untargeted GC–MS-profiling of metabolic alterations after dexamethasone treatment showed that dexamethasone had antithetical effects on the metabolic signature of the cells depending on growth conditions. The differentiated state of HEI-OC1 cells is better suited for elucidating metabolic changes induced by external factors. Dexamethasone treatment of differentiated cells led to an increase in intracellular amino acids and enhanced glucose uptake and β-oxidation in the cells. Increased availability of precursors for glycolysis and ATP production by β-oxidation stabilizes the energy supply in the cells, which could be assumed to be beneficial in coping with cellular stress. We found no negative effects of dexamethasone on the metabolic level, and changes may even prepare sensory cells to better overcome cellular stress following implantation surgery.

**Supplementary Information:**

The online version contains supplementary material available at 10.1007/s11306-021-01799-y.

## Introduction

Cochlear implants are widely used in restoring hearing in patients with severe or profound hearing loss (Naples & Ruckenstein, 2020). The prerequisite for this treatment is the presence of an intact cochlear nerve and a sufficient number of healthy spiral ganglia neurons that can be electrically stimulated by the implanted device (Ilberg et al., 1999; Kiefer et al., 2005). It is important to avoid inflammation and induced degenerative effects following insertion of the implant device because they may lead to direct or delayed hearing loss in cochlear implant patients (Eshraghi et al., 2007, 2015; Jia et al., 2013; Kamakura et al., 2018). Functional loss of the implant is caused by induced apoptosis in spiral ganglia neurons. Delayed hearing loss in patients with residual hearing in the low frequency range is the result of fibrosis and ossification destroying the microarchitecture of the inner ear, which may also occur months, or even years, after implantation (Jia et al., 2013; Linthicum et al., 2017; O'Leary et al., 2013; Quesnel et al., 2016). In addition, the latter leads to increased electrical impedance of the implanted electrodes, causing higher energy consumption and lowering the dynamic range (Kawano et al., 1998).

Synthetic glucocorticoids were first synthesized in 1957 and approved for medical use in 1961. Animal testing has shown that the use of glucocorticoids dampens foreign body reactions; consequently, the survival of hair cells and spiral ganglions is increased and fibrosis reduced (Eshraghi et al., 2007; Kuthubutheen et al., 2015; Malkoc et al., 2014). The first clinical pilot studies indicated that the use of glucocorticoids in cochlear implant surgery could prevent increased electrical impedance, improving preservation of the dynamic range (Bas et al., 2016; Kuthubutheen et al., 2017; Prenzler et al., 2018). These effects are attributed to the anti-inflammatory effect of glucocorticoids, which are agonists for the glucocorticoid receptor but have little affinity for the mineralocorticoid receptor (Liu et al., 2013). Because the glucocorticoid and mineralocorticoid receptors are expressed in many tissues, various effects are assumed for different parts of the body. For example, the systemic side effects of the glucocorticoid dexamethasone (Dex) are well studied and sometimes serious (e.g., reduced growth in children (Gibson et al., 1993), pancreatitis (Ksiądzyna, 2011), or the development of diabetes (Tappy et al., 1994). Therefore, the application of Dex in cochlear implant surgery is only done locally at the target structure in order to reduce systemic side effects, especially when the application is intended for a longer period of time. Thus, new application forms, such as cochlear implant electrode carriers releasing Dex, are currently under development (Liebau et al., 2019; Liu et al., 2016; Plontke et al., 2017).

The highest concentration of Dex is assumed to be elevated in the scala tympani and surrounding tissue, including the organ of corti and Rosenthal’s canal. Possible, local side effects to these sensory cells have not yet been part of metabolic investigations. Changes in pathways could lead to imbalances in energy homeostasis, potentially causing energy deficiency or the metabolization of intermediates that are relevant for cellular integrity and function. Thus, metabolic analysis is an important tool in elucidating aberrant changes induced in the whole metabolic network. Long-term observation studies in rats have revealed that continuous systemic application of Dex has a significant impact on the metabolome of neural cells in the brain (Dahabiyeh et al., 2020). To elucidate possible aberrant changes after Dex application in sensory cells of the inner ear, we performed a metabolic analysis to gain insight into alterations in cellular homeostasis in the presence of Dex.

We used the HEI-OC1 cell line derived from corti of organ explants, which express biomarkers that are characteristic of sensory cells in the organ of corti, as an in vitro model system. This cell line was established by Kalinec et al., and suggested for drug ototoxicity tests (Kalinec et al., 2003, 2016). The cells harbor an interferon-gamma-inducible promoter element controlling a temperature-sensitive mutant of the SV40 large T-antigen (Kalinec et al., 2016). This allows the ability to force the cells into a proliferative or differentiated state by incubating the cells at 33 °C or 39 °C, respectively. In the present study, we looked for changes induced by Dex in a hair cell-like cell line on a metabolic level in both growing conditions and compared them using GC–MS untargeted analysis. Metabolic analysis is an important tool in elucidating the aberrant changes induced in the whole metabolic network in addition to the intended effect of the drug used. Thus, it is an important tool in understanding side effects. The results of this study indicate antithetical effects on the metabolome after Dex treatment between proliferative and differentiated conditions in cells. The metabolic profiles of the cells grown under proliferative conditions exhibited little biovariability, whereas the metabolic profiles were more diverse under differentiated conditions. Our results indicate that Dex increases the intracellular abundance of energy metabolites.

## Methods

### Cell culture

HEI-OC1 cells were cultured in DMEM high Glucose Dulbecco’s Modified Eagle Medium (Life Technologies, Carlsbad, USA) with 10% FCS (Merck KGaA, Darmstadt, Germany) and 1% Penicillin G (Sigma-Aldrich, St. Louis, USA). Cells were incubated either at 33 °C and 5% CO_2_ or at 39 °C and 5% CO_2_ for three days to obtain cells in proliferative state and differentiated state respectively. Then cells were seeded 500,000 cells per 10 cm culture dish starting four groups i.e. a dexamethasone treatment and a control group for both cell conditions and cultured at proliferative and differentiated conditions in parallel (6 replicates for each condition). Additionally, plates were seeded in all cell conditions for cell counting (4 replicates). After 24 h 1 µL of a free dexamethasone solution in sterile water was added to the dexamethasone treatment conditions to obtain a final dexamethasone concentration of 10^−3^ M. 1 µL of sterile water was added to the control conditions. After further incubation for 3 days, 2 mL medium of each culture dish was shock frozen in liquid nitrogen and stored at − 80 °C. Dishes were washed with sterile 0.9% NaCl solution twice. Then 1.5 mL of cold methanol/water 9/1 (v/v) with 1 µg/mL ribitol + 1 µg/mL phenylglucose (internal standard for GCMS measurement) was added and cells were scraped from the dish bottom into the solution while culture dishes placed on ice. Afterwards the solution was shock frozen in liquid nitrogen and stored at − 80 °C. At the same time cells were counted in the plates seeded for cell counting. Prior to cell harvest, Cryo vials containing approximately 300 mg glass beads (diameter 425–600 μm; Sigma, Munich, Germany) were prepared. Sample extracts were filled on top of the glass beads and stored at − 80 °C until sample preparation.

### Sample preparation

Then samples were lysed using a Precellys bead-mill with 3 cycles at − 10 °C, 6500 rpm for 15 s and 10 s breaks in between runs. The mix was centrifuged (20,000×*g*, 4 °C, 15 min) and its supernatant subsequently dried in a vacuum centrifuge (Eppendorf, Hamburg, Germany). Dried samples were derivatised with 20 µL methoxyamine (20 mg/mL in Pyridine) at 28 °C for 90 min under constant shaking. Afterwards, samples were derivatized with MSTFA (N-Methyl-*N*-(trimethylsilyl)trifluoroacetamide; 37 °C, 30 min) and directly analyzed afterwards. QC samples were prepared by collecting and pooling 5 µL from each sample. The mixture was vortexed and aliquoted. A mixture of n-alkane mix (C_10_–C_40_, even; Neochema, Bodenheim, Germany) in hexane (GC-grade, Sigma-Aldrich, Taufkirchen, Germany) was used for retention index calculation.

### Instrumentation and data analysis

GC analysis was performed on an Agilent 7890 system coupled to an Agilent 5975C Mass sensitive detector (Agilent, Waldbronn, Germany). Samples (1 µL) were injected in splitless mode by a Gerstel MPS2 XL autosampler in randomized order and separated on a HP5-MS-capillary column (60 m × 0.25 m × 0.25 µm). Starting conditions for the temperature gradient was 80 °C, which was held for 3 min. Afterwards, the temperature was ramped for 5 °C/min for 49 min up to 325 °C. Temperature was held at 320 °C for 14 min. Total runtime was 66 min (Lagies et al., 2018).

Mass spectra acquired on an Agilent 5975C with an EI-source (Agilent, Waldbronn, Germany) were post-processed using AMDIS (Automated MassSpectral Deconvolution and Identification System; Version 2.71, Tobias Kind, Fiehnlab, California, USA) Version 2.71. For post-processing, Kovat’s retention indices were calculated from the n-alkane standard and applied to all the measured samples. Also, peaks were deconvoluted using AMDIS. Afterwards, common mass spectral features were matched, grouped and extracted by the online-tool Spectconnect (Styczynski et al., 2007). The resulting feature matrix with peak areas was annotated using a combination of NIST-database and in-house database. Acceptance criteria for annotation from database were retention index deviation < 5% and spectral similarity of fragments of  ≥ 75% (MSI identification level 1). Data was normalized to internal standard (ribitol or phenylglucose; whichever had a lower standard deviation of integrated peak areas across all samples) and cell count. Calculation of peak sums was performed by the summation of all peak areas of each sample. Subsequently, statistical analysis was performed using MetaboAnalyst 4.0. (Chong et al., 2018). Each variable was z-normalized prior to analysis. Detailed information of instrumentation and Data analysis is provided in Online Resource 5.

## Results

**Fig. 1:**
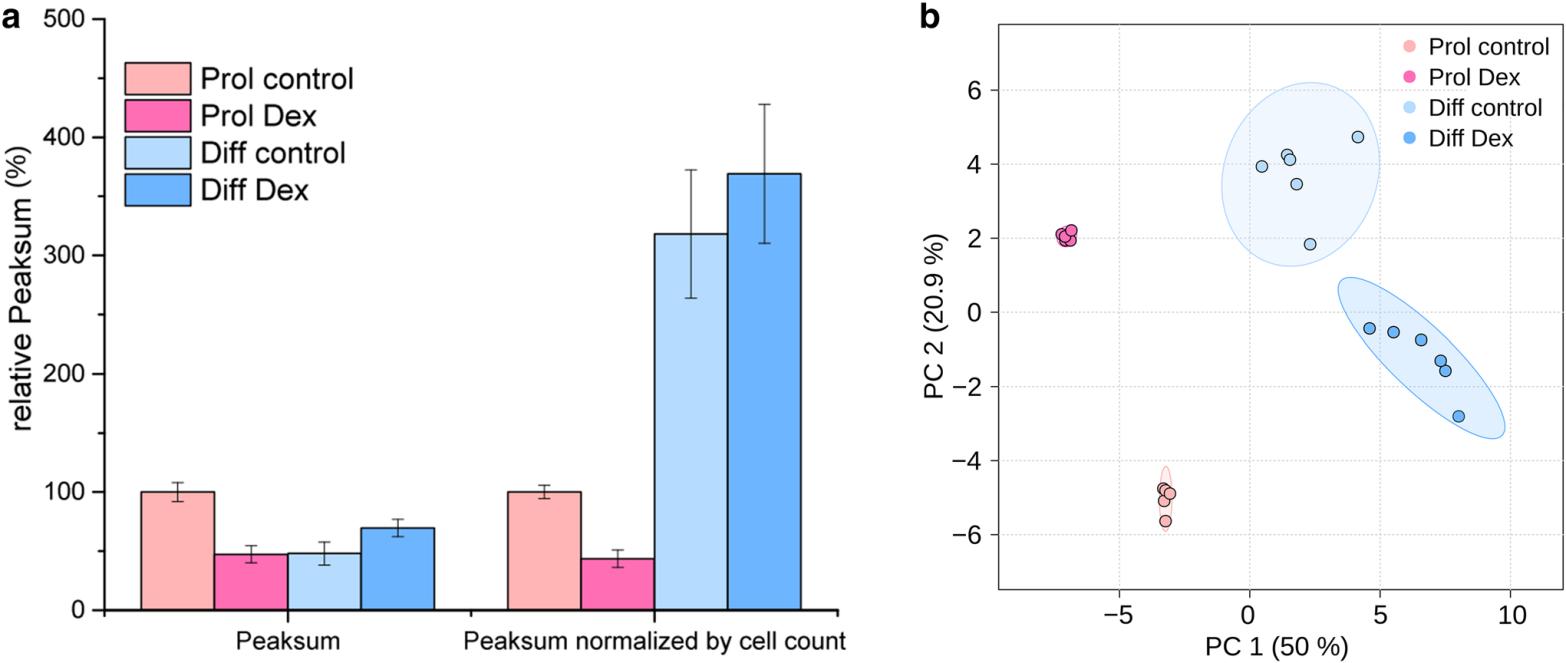
**a** Peak sum of peak areas in relation to the proliferation control (Prol control) group. Left, total peak sum in relation to the total amount of Prol control. Right, the ratio of peak sum to cell count in relation to the ratio of Prol control. Bars represent group averages. Error bars depict the standard deviation. **b** Principal component analysis plot of the four experimental conditions. PC1 separates cell condition (proliferative/differentiated), whereas PC2 separates Dex treatment and control. The QC shows that there was no shift during analysis. n(Prol control) = 5, n(Prol Dex) = 5, n(Diff control) = 6, n(Diff Dex) = 6.

For 221 mass spectral features, 71 corresponding metabolites were identified. Interestingly, we found considerable differences in peak sums between conditions (Table 1 and Fig. 1). The peak sum for Dex-treated cells cultivated under proliferative conditions was 50% lower on average compared to controls. For differentiated conditions, the peak sum was approximately 10% higher in Dex-treated cells than in controls. For this reason, the internal peak sum normalization was not feasible. Therefore, we used external normalization based on cell counting. Figure 1a depicts the peak sums of different experimental conditions and the peak sums normalized to total cell count per condition. Normalization for cell count revealed a much higher density of metabolites per cell under differentiated conditions.

**Table 1:** Peak sum and cell count for each group

Group	Peak sum	Cell count
Prol control	1.48 × 10^9^ ± 0.119 × 10^9^	12.6 × 10^6^
Prol Dex	7.00 × 10^8^ ± 1.08 × 10^8^	11.8 × 10^6^
Diff control	7.10 × 10^8^ ± 1.44 × 10^8^	1.7 × 10^6^
Diff Dex	1.03 × 10^9^ ± 0.109 × 10^9^	2.2 × 10^6^

*Prol control* proliferating condition, control, *Prol Dex* proliferating condition, dexamethasone, *Diff control* differentiated condition, control, *Diff Dex* differentiated condition, dexamethasone. Despite having 5- to 6-fold more cells in the proliferating groups, approximately similar total signal intensities were detected

A principal component analysis was performed using the online tool MetaboAnalyst (Fig. 1b) (Chong et al., 2018). Component 1 separates the two different cell conditions (i.e., proliferative or differentiated). The clusters on the left side of Fig. 1b are cells grown under proliferative conditions and the clusters on the right are cells grown under differentiated conditions. Component 2 separates control and Dex treatment. The clustering of both groups under proliferative conditions is very narrow, showing little room for biological variation when cells are forced into the proliferative state, which highlights its artificial character.

**Fig. 2:**
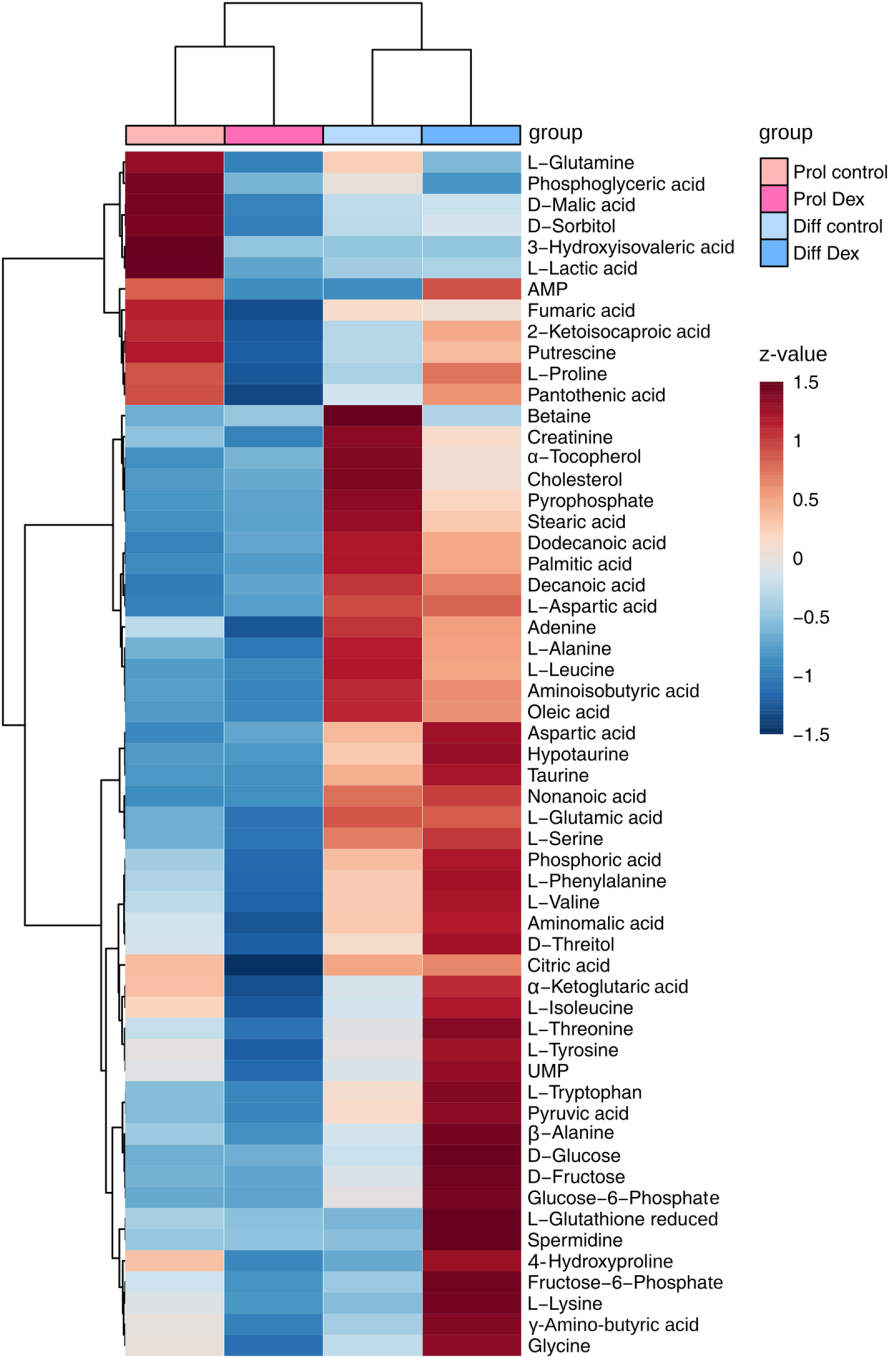
Hierarchical clustering heat map of all confirmed intracellular metabolites after polar extraction. Group averages for each metabolite were z-normalized and depicted in a color code. Dendrograms were generated with Euclidean distance measure and Ward’s clustering algorithm. n(Prol control) = 5, n(Prol Dex) = 5, n(Diff control) = 6, n(Diff Dex) = 6

All confirmed metabolites analyzed in the cells are shown in the heat map in Fig. 2. Normalized peak data for cell and medium analysis are available in Online Resource 1 and 2. Most metabolites had relatively higher concentrations in the differentiated state than the proliferative state. Pathway mapping with bar charts for key metabolites of each condition of the main energy pathways in medium and lysate and each condition are available in Online Resource 6 and 7. A horizontal line above two bars represents a significant difference with ANOVA post-hoc test. The underlying date for the bar charts is available in Online Resource 8 and 9. Results for ANOVA post-hoc testing are in Online Resource 3 and 4.

**Fig. 3:**
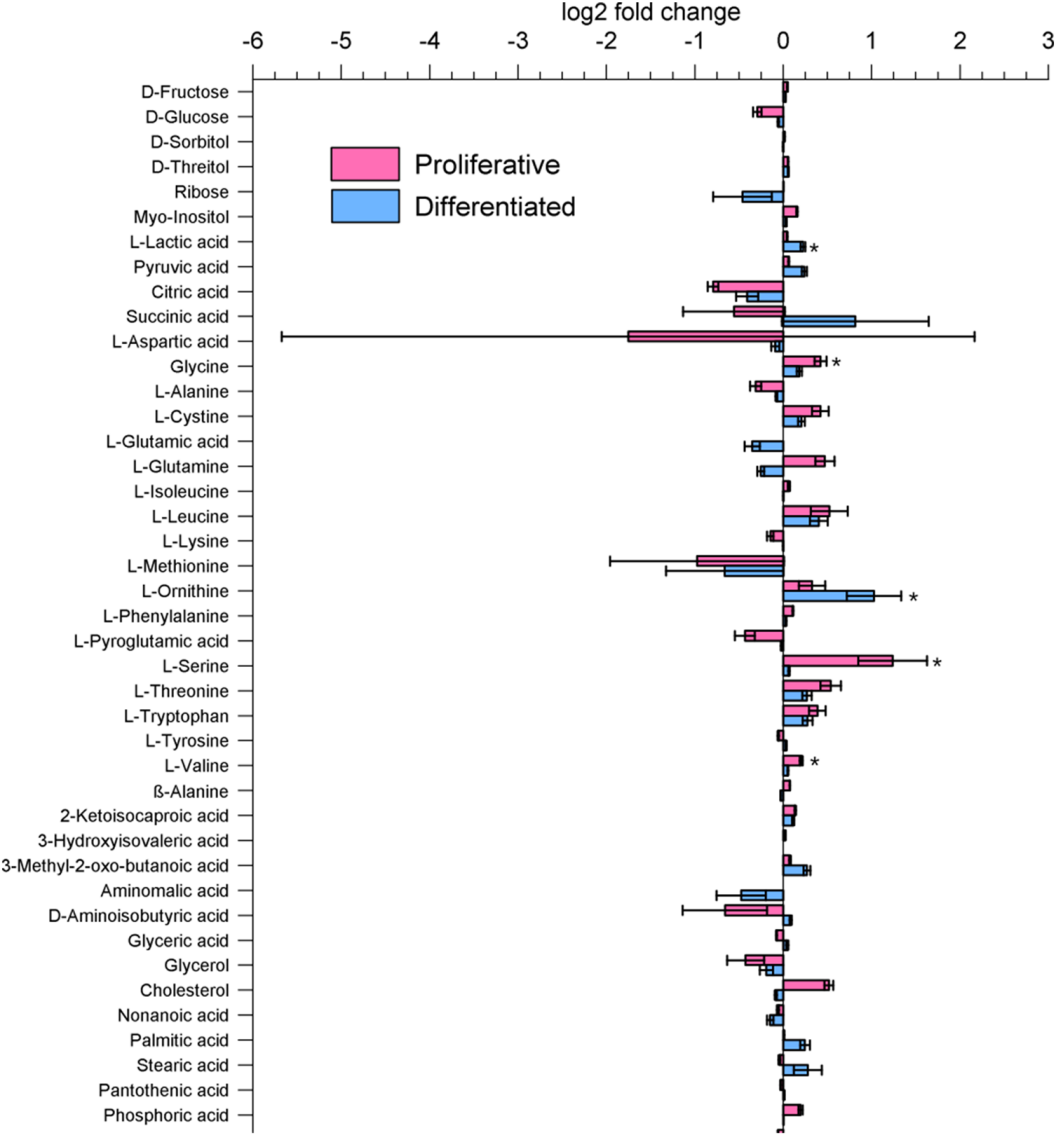
Log2 fold changes of metabolites in medium due to Dex treatment in different conditions (proliferative/differentiated). The asterisk (*) indicates a significant difference (p < 0.05) in the fold change within the group by ANOVA with Fisher's least significant difference post-hoc test. n(Prol control) = 6, n(Prol Dex) = 6, n(Diff control) = 6, n(Diff Dex) = 6

**Fig. 4:**
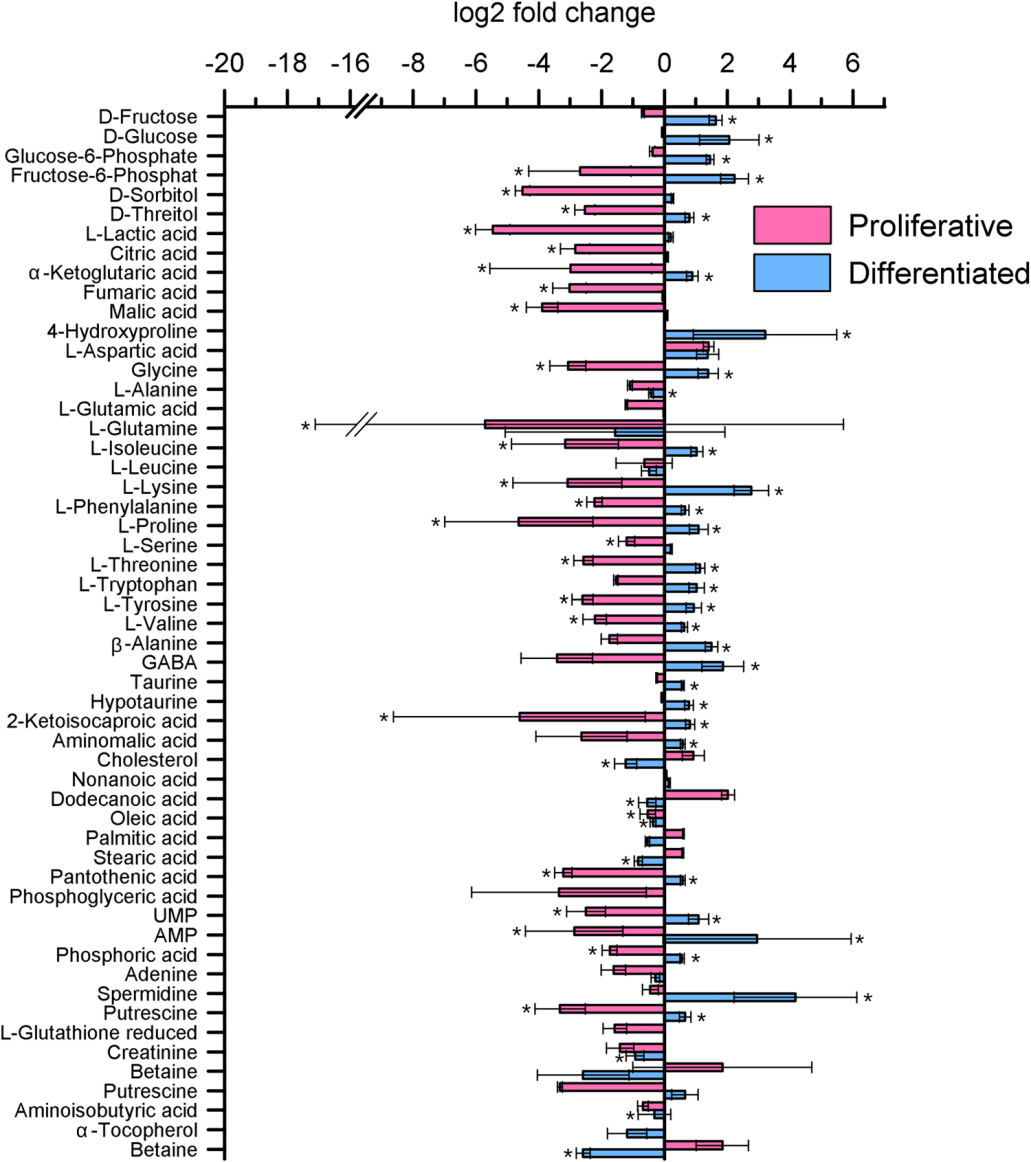
Log2 fold changes of metabolites in cells due to Dex treatment in different conditions (proliferative/differentiated). The asterisk (*) indicates a significant (p < 0.05) difference in the fold change within the group by ANOVA with Fisher's least significant difference post-hoc test. n(Prol control) = 5, n(Prol Dex) = 5, n(Diff control) = 6, n(Diff Dex) = 6

Figures 3 and 4 depict the fold changes between Dex exposition and controls in both growing conditions in medium and cells, respectively. Data are available in Online Resources 3 and 4. Direct comparison of control groups without Dex treatment revealed that intracellular concentrations of sugars (i.e., fructose, glucose, and glucose-6-phosphate), except lactate, were significantly higher under differentiated conditions. The concentration of lactate was significantly lower in cells grown under differentiated conditions, suggesting a lower glycolysis rate in cells growing in differentiated conditions than in proliferating cells. The higher proliferation rate of the cells grown under proliferative conditions is reflected in the significantly lower amounts of intracellular fatty acids, such as palmitic and stearic acid, and membrane components, such as cholesterol. Glyceric acid was also increased in the medium (Fig. 3), which supports the assumption that β-oxidation rates are also upregulated. The intracellular concentrations of TCA-intermediates citric acid and a-ketoglutaric acid are not significantly different between growth conditions. In addition, fumaric acid and malic acid are significantly more abundant in proliferating cells, suggesting reduced energy generation from oxidative phosphorylation, which is supplemented by other energy sources such as β-oxidation, glycolysis and its subsequent lactate formation.

Examining the fold changes in metabolites between the control and Dex-treated groups revealed antithetical effects of Dex on cell metabolism depending on the growth condition (Fig. 4). Most metabolites are decreased under proliferative conditions during Dex incubation, whereas most metabolites are increased under differentiated conditions after Dex treatment compared to controls.

### Metabolic alterations in proliferating HEI-OC1 cells

As already mentioned above, the total metabolite count per cell was substantially different based on cell status. In proliferating Dex-treated cells, we measured a significant decrease in lactate levels, even when compared to the differentiated groups. However, all detected sugars except fructose-6-phosphate were depleted in proliferating groups for both Dex-treated cells and controls. Within proliferating groups, no significant differences were detected between the amounts of sugars, with the sole exception of fructose-6-phosphate, which was significantly reduced in Dex-treated cells. A lower concentration of glucose in the medium of the Dex-treated group compared to non-treated cells indicates higher glucose uptake induced by Dex.

Increased glycolysis activity would normally lead to accumulation of lactate, which is not seen in proliferating Dex-treated cells under anaerobic conditions. Interestingly, lactate accumulated in the medium of the proliferating groups with a slight trend towards higher amounts of lactate in the medium of Dex-exposed cells. This suggests an upregulation of lactate efflux in proliferating cells, especially in the Dex-treated group. The low levels of intracellular lactate in proliferating Dex-treated cells go along with depleted amounts of all detected TCA intermediates, including citrate, α-ketoglutaric acid, fumarate, and malate. Considering that the formation of citrate is the rate-limiting step in the TCA-cycle, we assume that the production of energy by oxidative phosphorylation is upregulated under the influence of Dex in proliferating conditions.

All ketogenic amino acids and potential ketogenic amino acids, which may be converted to acetyl-CoA, were concentrated at lower levels. Keeping in mind that glycolytic activity is reduced, energy provision must be supplemented by other pathways, such as the TCA-cycle. Thus, we assume amino acids are fed into the TCA-cycle, resulting in the overall low levels of metabolites (i.e., TCA intermediates and amino acids). In addition, pantothenic acid, a precursor of coenzyme A and a cofactor for acyl-carrier protein, was reduced significantly, approximately fivefold, in Dex-exposed cells. This goes along with depleted free fatty acids and cholesterol for both proliferating groups with a tendency of slightly higher amounts of fatty acids in Dex-treated cells, which may indicate an increased metabolism of fatty acids by β-oxidation. At the same time, we observed significantly increased glyceric acid levels in medium for both proliferating groups compared to the differentiated groups, suggesting an increased efflux of free glyceric acids due to increased β-oxidation. The cholesterol concentration was non-significantly altered by Dex incubation in cells and medium. However, in both proliferative groups, the amount of cholesterol was significantly lower than under differentiated conditions. A high cell proliferation rate decreases the cholesterol membrane content. Thus, more cholesterol is needed to maintain membrane fluidity, which probably results in cells actively importing cholesterol from the medium or passive uptake of cholesterol into membranes.

### Metabolic alteration in differentiated HEI-OC1 cells

The metabolome of the differentiated cells differed widely from that of proliferating cells. The cells growing without the active virus protein generally had a higher amount of (energy) metabolites, predominantly the precursors for glycolysis and β-oxidation. The sugars fructose-6-phosphate, glucose-6-phosphate, glucose, and fructose were greatly increased. We found the highest amounts of sugars in differentiated Dex-treated cells compared to all other groups. As in proliferating Dex-treated cells, a slight trend of increased glucose uptake from the medium was also apparent for the differentiated Dex group. Despite the high availability of sugars in the cells, the rate of glycolysis did not appear to be elevated in the differentiated groups. The levels of lactate, pyruvate, and TCA intermediates citric acid, fumaric acid, and malic acid were not significantly different, indicating no change in oxidative phosphorylation. Most of the detected fatty acids and cholesterol were significantly reduced in Dex-incubated cells compared to controls. The opposite was true for essential amino acids, ketogenic amino acids, and most amino acids that can feed into the TCA cycle, which were increased in Dex-treated cells. This finding suggests a higher energy uptake by means of increased import of metabolites by the cells under the influence of Dex. The assumption is also supported by the aforementioned increase in sugars in cells and the decreased glucose in the medium of Dex-treated groups. However, the levels of ketogenic and essential amino acids in medium remained mostly unchanged, which supports the assumption of diminished consumption of these amino acids.

Decreased amounts of fatty acids may also point to increased energy provision by the generation of acetyl-CoA with β-oxidation. However, one of the essential precursors in acetyl-CoA synthesis, pantothenic acid, was increased in Dex-exposed cells, suggesting that acetyl-CoA is not increased, but decreased. This suggests an increased turnover of acetyl-CoA. Dex-treated cells had significantly lower cholesterol content than cells from the respective control groups. At the same time, the amount of cholesterol in the medium seemed to be unaffected by Dex treatment, which suggests that the import of cholesterol into the cells is not altered. Therefore, Dex appears to have an attenuative influence on cholesterol synthesis.

## Discussion

HEI-OC1 cells were incubated under proliferative or differentiated conditions. Each growth condition was treated with Dex and compared to its respective control group. Untargeted GC–MS profiling revealed 70 significantly altered metabolites in ANOVA testing. Our results show that the active temperature sensitive SV40 large T antigen gene that forces cells into the proliferative immortal state leads to massive overall metabolite depletion, in addition to rapid proliferation. The analysis also revealed that cells growing under forced proliferative conditions feature little biovariability. This depicts a very stable, yet artificial, metabolism in which cells increase their energy production to provide enough energy for continuous mitosis. In addition, the active virus protein inverted the effect of Dex incubation on most metabolites, leading to antithetical effects on the metabolome between the proliferative and differentiated conditions. One has to keep in mind the artificial metabolism state when using this cell line for conducting drug ototoxicity tests under proliferative conditions. For this reason, we think that HEI-OC1 cells growing without the influence of the virus protein represent a more natural model for metabolomics experiments.

Rapidly proliferating cells are usually characterized metabolically by increased glycolytic activity leading to lactate accumulation (Cori & Cori, 1925; Warburg et al., 1927; Young, 2013). Interestingly, this was only seen in the proliferating control group, not in Dex-treated cells, which generally had lower amounts of metabolites, including lactate. The medium had highly increased levels of lactate for Dex-treated cells. As intracellular metabolite composition points to increased glycolysis in these cells, Dex seems to force the efflux of lactate.

The increase in lactate production with depleted amounts of precursors for glycolysis (i.e., sugars and sugar phosphates_ in proliferating cells implies highly elevated glycolytic activity (Ippolito et al., 2019). In concert with increased glycolytic activity, oxidative phosphorylation also appears to be upregulated because of increased amounts of malate und fumarate. For some immortal cell lines, increased oxidative phosphorylation activity has already been observed (Moreno-Sánchez et al., 2007; Weinberg & Chandel, 2015).

Levels of free fatty acids were not significantly altered between the proliferating control and Dex-treated groups. However, the amount of pantothenic acid was 10-times lower in Dex-exposed cells, suggesting increased usage for the synthesis of acetyl-CoA. At the same time, no significant difference in pantothenic acid was found in the medium across all four groups, indicating no altered uptake or release from the cells.

In cell models, Dex exposure is usually associated with increased uptake of nutrients (Dyczynski et al., 2018). In HEI-OC1 cells, a tendency for increased uptake of glucose was seen for Dex-treated cells in both conditions. All detected intracellular sugars were significantly more abundant in differentiated Dex-treated cells than the respective controls. In addition, an intracellular increase in essential amino acids (i.e., l-threonine, l-isoleucine, l-lysine, and l-tryptophan) was measured in the differentiated Dex-treated group. However, the amounts of amino acids in medium remained unchanged. As the intracellular TCA metabolites did not differ in the differentiated group, reduced introduction of amino acids into the TCA cycle in the Dex group seems likely. Furthermore, due to increased uptake and, thus, increased availability of glucose for glycolysis, there would be a sufficient supply of pyruvate for oxidation during the TCA cycle. Increased protein catabolism may be an alternative cause of higher amounts of free amino acids, which would have to be evaluated as a negative effect of Dex treatment for longer duration. However, this effect has mostly been reported for skeletal muscle cells (Moreno-Sánchez et al., 2007; Dyczynski et al., 2018; Malkawi et al., 2019), though it has also been postulated to occur in brain tissue (Malkawi et al., 2019). As we found no observable difference in the rate of oxidative phosphorylation but increased availability of sugars and apparent increase in β-oxidation, the cells have plenty of sources for the generation of ATP. When there is no need for the cells to use amino acids for ATP generation, amino acids, particularly essential amino acids are available for protein synthesis. On the other hand, one could hypothesize that decreased protein biosynthesis as a factor underlying increased amino acid levels in Dex-exposed cells. Further proteomic analysis may be necessary to prove stable protein turnover in Dex-exposed cells. Flux analysis with C^13^-labeled amino acids could be used to track amino acids and quantify changes in their introduction into the TCA cycle during Dex exposure.

Fatty acids, such as palmitic acid, stearic acid, and oleic acid, are significantly reduced in the differentiated Dex-treated group. Therefore, the energy production is probably shifted toward β-oxidation by Dex incubation. This oxidation process yields further ATP, as well as the final product, acetyl-CoA, which is also a precursor for the TCA cycle. The cholesterol concentration was significantly reduced in the cells, whereas the concentration in the medium remained unchanged. Other studies have shown a suppressive effect of glucocorticoids on cholesterol synthesis (Johnston et al., 1980; Picard et al., 1980). Our data support the assumption that this may also be true for HEI-OC1 cells.

A limitation of the present study is the short incubation with Dex. The incubation time had to be limited to obtain confluent cells at the time of sampling in order to exclude effects on the metabolome induced by high cell density. However, local Dex treatment after cochlear implant surgery is intended for several weeks. Therefore, we cannot completely rule out further Dex-induced changes on the metabolomic level that are not seen during the first few days of incubation.

An increased amount of available energy in the cell has been associated with positive effects on regeneration processes. A previous study showed that regeneration of damaged axons is positively influenced by enhancement of the local energy supply by stimulating mitochondrial transport to the injured part of the axon (Han et al., 2020).

Photobiomodulation with near infrared light has been suggested to restore ATP production by photo dissociation of nitrous oxide (NO) from cytochrome-c oxidase as part of the mitochondrial respiratory chain, accelerating the rate of respiratory chain activity (Hashmi et al., 2010). It has been hypothesized that, in the case of mitochondrial dysfunction due to cell stress, cytochrome-c oxidase is inhibited by competitive binding between NO and O_2_ (Bartos et al., 2016). In postnatal rat neurons, mitochondrial dysfunction induced by potassium cyanide can be prevented based on increased cell vitality when cells were pretreated with 670 nm and 830 nm light exposure (Wong-Riley et al., 2005).

With increased availability of glucose and substrates for glycolysis, increased ATP production by β-oxidation, decreased consumption of amino acids, and subsequently increased amounts of (essential) amino acids for protein synthesis, we propose that there are no disadvantageous effects on metabolic changes with regard to energy metabolism in sensory cells of the inner ear after local application of Dex for a limited time after implantation surgery. Furthermore, the results of our metabolomic study suggest that the effects induced by Dex on metabolic changes are beneficial in coping with cellular stress following surgical trauma.

## Electronic supplementary material

Below is the link to the electronic supplementary material.

Electronic supplementary material 1 (XLSX 34 kb)

Electronic supplementary material 2 (XLSX 42 kb)

Electronic supplementary material 3 (XLSX 12 kb)

Electronic supplementary material 4 (XLSX 14 kb)

Electronic supplementary material 5 (DOCX 16 kb)

Electronic supplementary material 6 (PNG 770 kb)

Electronic supplementary material 7 (PNG 853 kb)

Electronic supplementary material 8 (XLSX 31 kb)

Electronic supplementary material 9 (XLSX 32 kb)
