# Supplementary material for: Metabolic reprogramming of inner ear cell line HEI-OC1 after dexamethasone application
Source: Metabolomics. 2021 May 24;17(6):52. doi: 10.1007/s11306-021-01799-y (PMC8144088; doi:10.1007/s11306-021-01799-y)
Supplement: Supplementary file 5 — Electronic supplementary material 5 (DOCX 16 kb) [file 11306_2021_1799_MOESM5_ESM.docx]

1. GC-MS settings::

- Injection mode: Pulsed splitless
- Injection Pulse Pressure: 25 psi
  until 0.8 min
- Purge Flow to Split Vent: 100 mL/min at 1.5 min
- Inlet temperature: 230 °C
- Inlet pressure: 19.035 psi
- Septum purge flow: 3 mL/min
- Column gas flow: 1mL/min
- Column gas pressure: 19.305 psi
- Post run flow: 1 mL/min

1. Data processing:

**AMDIS Analysis Settings:**

*Identification:*

- Minimum Match Factor: 60
- Multiple identifications per compound
- Show standards
- Only reverse search
- Type of analysis: Simple

*Instrument:*

- Low m/z: 50
- High m/z: 800, Threshold: Off
  Scan direction: High to Low
- Data File Format: Agilent Files
- Instrument Type: Quadrupole

*Deconvolution:*

- Component width: 12
- Omit m/z
- Adjacent peak subtraction: One
- Resolution: Medium
- Sensitivity: Medium
- Shape requirements: Medium

*Library:*

MS libraries/RI data: Target Compounds Library

*QA/QC:*

- Solvent tailing: 84 m/z
- Column bleed: 207 m/z

*Scan Sets:*

- Number of sets: 3
- Scan Set 1
- Start: Start
  End: 4.8 min
  Low m/z: 15
  High m/z: 300
- Scan Set 2
  Start: 4.8 min
  End: 60 min
  Low m/z: 29
  High m/z: 350
- Scan Set 3
  Start: 60 min
  End: 9999 min
  Low m/z: 40
  High m/z: 700

Filter:

- Enable Filters

**Spect Connect Settings:**

- Elution threshold: medium (1min)
- Support threshold: medium (>= 75 % of samples)
- Similarity threshold: medium (80 % match)

**MetaboAnalyst Settings:**

- The tool “statistical analysis” was used. Peak intensity tables “Samples in columns (unpaired) were uploaded and submitted for processing.
- Data Filtering: none
- Sample Normalization: none
- Data transformation: none
- Data scaling: Auto scaling
