# Supplementary figures and images for: Metabolic reprogramming of inner ear cell line HEI-OC1 after dexamethasone application
Source: Metabolomics. 2021 May 24;17(6):52. doi: 10.1007/s11306-021-01799-y (PMC8144088; doi:10.1007/s11306-021-01799-y)

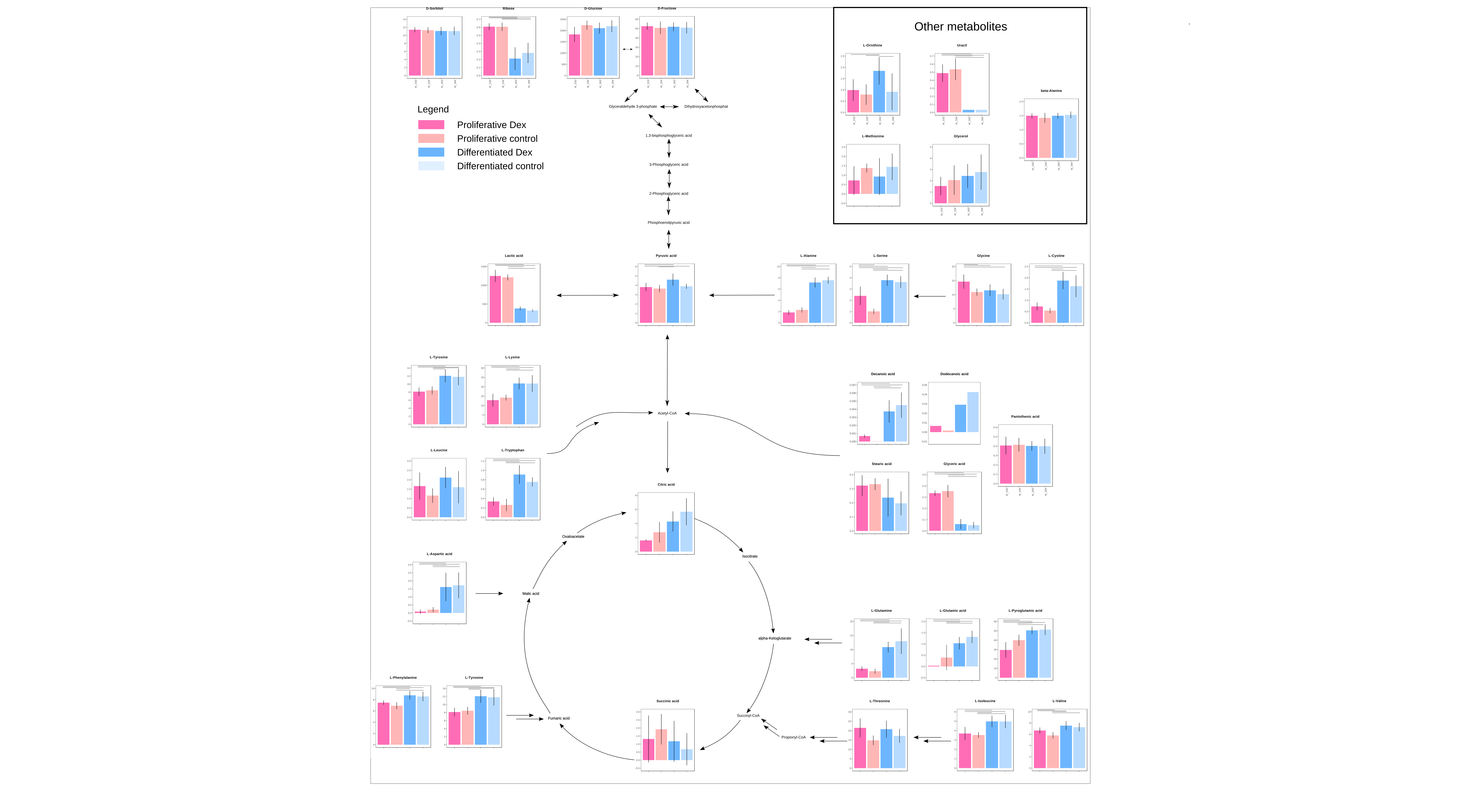

Supplement: Supplementary file 6 — Electronic supplementary material 6 (PNG 770 kb) [file 11306_2021_1799_MOESM6_ESM.png]

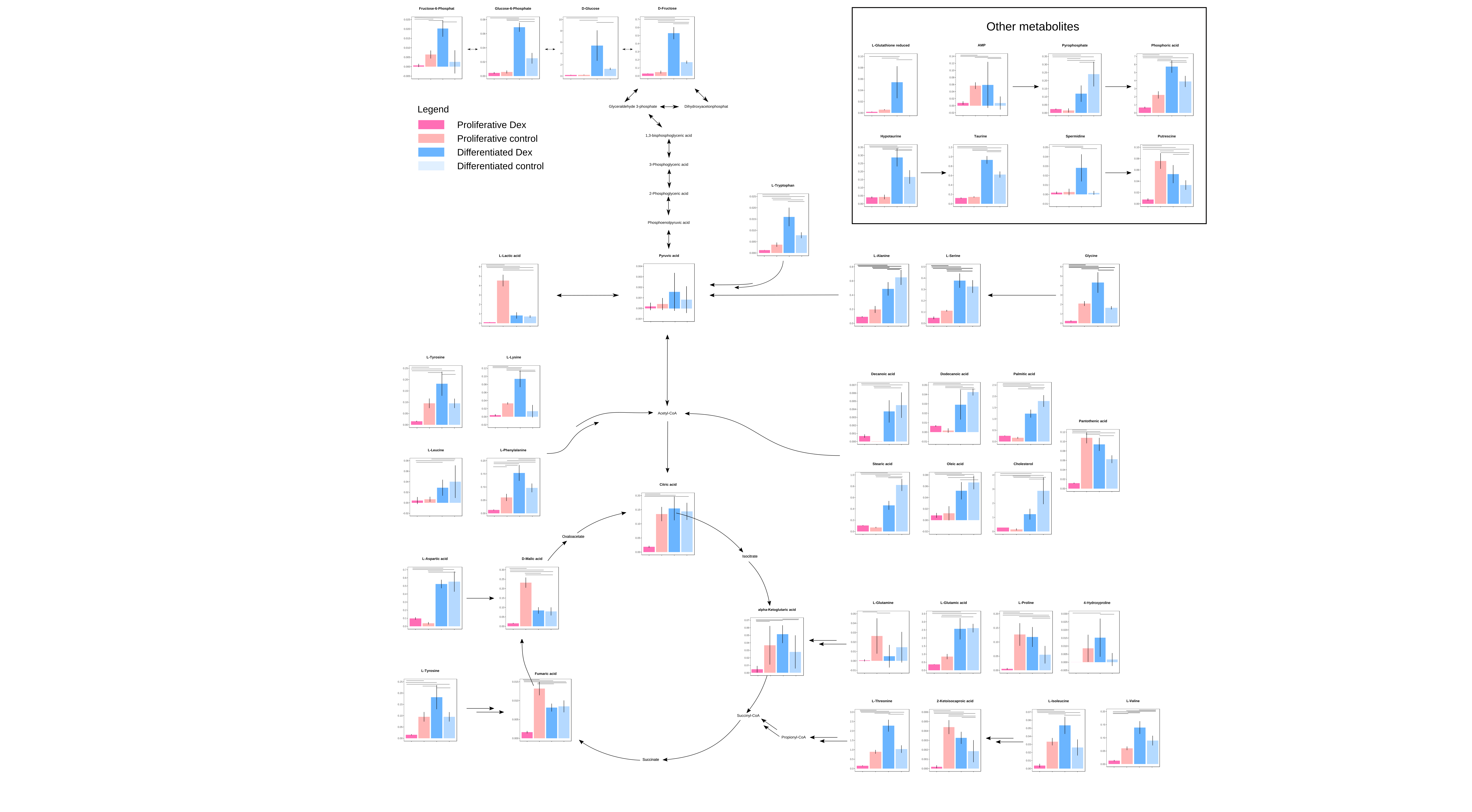

Supplement: Supplementary file 7 — Electronic supplementary material 7 (PNG 853 kb) [file 11306_2021_1799_MOESM7_ESM.png]
